# Identification and Characterization of a Protease Producing *Bacillus cereus* Strain From Tannery Waste for Efficient Dehairing of Goat Skin

**DOI:** 10.1155/bmri/7639181

**Published:** 2025-01-06

**Authors:** Md. Ekhlas Uddin, Md. Ramjan Sheikh, Md. Asaduzzaman, Sohel Ahmed, Sukalyan Kumar Kundu, Abu Ali Ibn Sina

**Affiliations:** ^1^Department of Biochemistry & Molecular Biology, Gono Bishwabidyalay, Savar, Dhaka, Bangladesh; ^2^Department of Pharmacy, Jahangirnagar University, Savar, Dhaka, Bangladesh; ^3^Department of Biochemistry & Molecular Biology, Jahangirnagar University, Savar, Dhaka, Bangladesh; ^4^Center for Personalized Nanomedicine, Australian Institute for Bioengineering & Nanotechnology (AIBN), The University of Queensland, Brisbane, Queensland, Australia

**Keywords:** bioremediation, ecofriendly environment, identification, leather residues, molecular characterization, protease producing bacteria

## Abstract

Environmental pollution has been a significant concern for the last few years. The leather industry significantly contributes to the economy but is one of Bangladesh's most prominent polluting industries. It is also responsible for several severe diseases such as cancer, lung diseases, and heart diseases of leather workers because they use bleaching agents and chemicals, and these have numerous adverse effects on human health. The study was aimed at isolating, identifying, and molecularly characterizing bacteria that produce protease enzymes that are highly capable of dehairing goat hide. Several attempts were made to isolate and identify protease-producing bacterial strains from different soil samples of tannery wastes. Initially, a total of four isolates were selected from tannery soil. After the different phenotypic and morphological characterization, one isolate (BS2) showed Gram-positive, rod-shaped, and spore-forming characteristics and could produce novel hair-degrading protease enzymes. The growth profile and protease activity of the bacteria at 37°C were observed; a basal level of extracellular protease increased with time. The enzyme's proteolytic activity was measured, and the unit of enzyme activity of the enzyme sample was 18.1. The ExPASy server (ProtParam) tool was used to estimate the physicochemical characteristics of the proteins and found molecular weight (MW) (7375.94 Da), aliphatic index (71.56), instability index (II, 80.63), Grand Average of Hydropathy (GRAVY) (−0.231), and isoelectric point (11.41). The protein–protein interactions (PPI) networks were generated using the Search Tool for the Retrieval of Interacting Genes (STRING) database and Cytoscape software. The PSIPRED v.4.0 and SAVES v.6.0 programs were used to determine the secondary and three-dimensional assembly of the selected protein. They found alpha helix (16, 25.00%), extended strand (6, 9.38%), beta-turn (5, 7.81%), and random coil (37, 57.81%). DNA isolation and purification were carried out, and the purity ratio was ~2.17 at 260 and 280 nm. Polymerase chain reaction (PCR) for amplifying the 16S rRNA gene was conducted, and the isolate was authentically recognized as *Bacillus cereus* (BS2) based on morphological, biochemical, and molecular analyses. The quantitative assessment has shown that 40 mL of culture centrifugation could dehair 2 × 1 cm of goat leather sample in 9 h. This potential bacterial strain can be used in the leather industry as an ecofriendly alternative to chemical dehairing, which can reduce environmental pollution and the risk of severe diseases among leather industry workers.

## 1. Introduction

The leather industry was established in Bangladesh on a large scale, mainly in the 1970s, and is one of the most extensive industries in Bangladesh. The leather-based items include garments, footwear, belts, bags, wallets, suitcases, and decorative items developed depending on the leather industries. Leather industries have played a crucial part in our national economy, gaining many foreign remittances. Bangladesh earned $1.01 billion by exporting leather, leather footwear, and leather items, the only billion-dollar export earnings after apparel products in the fiscal year 2019 [1].

Most leather industries use chemicals for leather dehairing, which poses a massive threat to the environment and ecosystem [2]. A recent estimate showed that approximately 60 × 10^3^ tons of raw hides and skins were treated in these industries every year, and nearly 90,000 L of untreated discharge daily, which contains heavy metals, resulted in the death of the Buriganga River [3]. In recent times, enzymatic dehairing has shown great promise as an ecofriendly alternative to the conventional chemical process [4]. Dealing with unnecessary proteins using ecofriendly and cheapest methods is the central requirement in leather factories. Enzymes such as lipase, keratinase, and protease are used in the leather business to soften fibers, enhance quality, and offer substitutes for the chemical process. The proteolytic processing of skins involves the degradation of the joining materials holding the hair to the hide to be taken out without any damage [3].

Protease enzymes can be synthesized from various plants, microorganisms, and animals. Several micro-organisms, including bacteria, yeasts, and fungi, have been analyzed simultaneously to generate multiple biocatalysts for industrial utilization [5]. The application of microorganisms to manufacture the proteolytic enzyme has several advantages. Nowadays, bacterial proteases are incorporated into fish/shrimp feed for bioremediation processes and are also aquaculture probiotic agents [6]. Proteases are used in dehairing and dewooling leather to improve its qualities (cleaner, healthier surface, softener leather, and fewer spots) [7].

Proteases also have hydrolytic activity that hydrolyzes peptide bonds between proteins, and they have practical applications in the pharmaceutical and manufacturing sectors [8]. Alkalophilic microbes are commonly distributed in natural environments without the constraint of alkalinity [9, 10]. Several microbial enzymes are used as organic catalysts on an industrial scale for their particular interests in the processing industry [11]. Enzymes have been used in heterogeneous applications from industrial sectors to household products and are valuable in leather processing [12]. Thus, enzyme-based dehairing activities using proteases help to decrease or even prevent that chemical and offer tremendous ecological benefits [13]. Therefore, it is significant to search for proteases with profitable resources for industrial sustainability from bacterial origins [14]. Microorganisms represent an attractive enzyme origin due to their vast biochemical variety and sensitivity to biogenetic manipulation [15]. In addition, it is used to improve the quality of the leather products. Electrode substance technology improves environmental conservation by converting industrial rubbish into viable materials for energy preservation. It emphasizes the possibility of using waste-derived carbon substances to sustainably address global thermal storage demands [16]. However, this technology has limitations and challenges, yet it is ripe alongside the potential of converting trash into valuable materials in energy storage. Alternately, enzymes from microbes are known to be special enzymes extracted from numerous microorganisms, such as bacteria, yeast, and fungi, specifically for utilization in industries on commercial grades [17]. This is important considering Bangladesh is still growing its industrial manufacturing sector [18]. This study's objectives were to isolate, identify, and characterize the bacteria-producing protease enzymes from the soil of tannery waste. This study's results are proposed as an alternate source of protease enzyme contributing to the tanner industry, especially in the dehairing phase.

## 2. Methodology

### 2.1. Sample Collection and Experimental Site

The whole study was done under the laboratory of the Department of Microbiology and Biochemistry, Gono Bishwabidyalay, Savar, Dhaka, partially in association with Invent Technologies Ltd, Dhaka, and IFRB division, BAEC, Ganakbari, DEPZ, Savar, Dhaka. Soil samples were obtained from various parts of the ternary industrial area of Hemayetpur, Savar, and Dhaka, and they were labeled with the site name and collection date. After collection, samples were immediately transferred to the respective department for processing.

### 2.2. Sample Processing and Isolation of the Microorganisms

After adjusting the pH, serial dilutions were done, followed by the spread plate method to see the growth of different microorganisms in sleeted samples [19]. Tenfold serial or stepwise dilution of the soil samples was prepared with normal saline. After finishing serial dilution, the diluted samples were spread in the respective nutrient agar plates, and the diluted samples were spread using spread plate technique. All the culture plates were then incubated at 37°C for 24–48 h. Finally, the plates showing colonies were counted and noted for further study. Therefore, an isolated colony was picked from the agar plate, striking out over the plate using close analogous streaks, and incubated at 37°C for 24 h.

### 2.3. Maintenance of Pure Culture

The colonies from nutrient agar were then streaked onto the *Bacillus cereus* agar (selective media), incubated at 37°C for 24 h, and then stored at 4°C until use [20].

### 2.4. Bacterial Identification

#### 2.4.1. Morphological Characterizations of Bacteria

Nutrient agar was prepared and autoclaved at 121°C and 15 psi. The media was dispensed into sterile plates while liquid and left for a while to solidify. Using the sterile technique, a nutrient agar plate was streaked by a 24-h-old pure cultures loop with an inoculating loop employing the three-quadrant streak plate method to obtain isolated discrete colonies. The culture plates were then incubated at 37°C for 24 h. After incubation, the growth arrangements of the bacteria were evaluated for size, pigmentation, form, margin, elevation, and texture [21].

#### 2.4.2. Microscopic Observation by Gram Staining Technique

After the slides were prepared, samples were reviewed under an ultramicroscope using an oil emulsion scheme with 100x magnification power. The results were recorded as purple for Gram-positive or pink for Gram-negative (−ve) bacteria [22]. Gram staining and spore staining features of microorganisms were studied microscopically for every sample individually.

### 2.5. Biochemical Characterization of Bacteria

Biochemical tests of the isolates were done, including catalase test, oxidase test, indole production test, citrate utilization test, Methyl Red (MR) test, Voges–Proskauer (VP) test, lactose test, sucrose test, casein hydrolysis, gelatin hydrolysis, starch hydrolysis, urease test, carbohydrate fermentation, urease test, and nitrate reduction test.

### 2.6. Enzymatic Extraction From Bacterial Isolates

For the protease production studies, bacteria were inoculated into Luria–Bertani (LB) medium prepared in seawater and maintained at room temperature (25°C) for 24 h on a shaker (140 rpm); after this period was completed, it was centrifugated at 10,000 rpm and 4°C for 15 min [23]. The clear supernatant was used for the dehairing experiment, and cell-free supernatant was obtained for the enzyme assay test.

### 2.7. Analysis of the Proteolytic Activity on Azocasein Assay

The azocasein test measured the proteolytic activities described by the Kreger and Lockwood method, 1981. Here, azocasein is used as a substrate.

### 2.8. Determination of Growth Profile and Protease Activity

Bacterial growth was measured at different time intervals at 37°C temperature. Consequently, bacteria were cultured in nutrient broth (NB) at 37°C, and fresh culture was taken at different time intervals (4, 6, 8, 10, 12, 13, 14, 15, 16, 18, 20, 22, 24, 26, 28, 30, and 32 h); then, the absorbance was measured at 600 nm.

Simultaneously, the protease activity was assessed, followed by the same techniques for the bacterial growth profile, and then absorbance was measured at 440 nm.

### 2.9. Bioinformatics Analysis for the Characterization

#### 2.9.1. Physicochemical Characterization

Molecular identification confirmed that the identified bacterium was *B. cereus*. The ExPASy translate and in silico translate tools were used to estimate the amino acid sequences [24, 25]. Moreover, ExPASy's ProtParam program [26] was used to demonstrate the physicochemical factors of the desired protein.

#### 2.9.2. Determination of Subcellular Localization

The selected protein's subcellular localization was analyzed using the prediction of subcellular localization tool Version b (PSORTb) [27], protein subcellular localization predictor (PSLpred) server [28], hidden Markov model for topology prediction (HMMTOP) [29], and deep learning transmembrane hidden Markov model (DeepTMHMM) [30] servers.

#### 2.9.3. Protein–Protein Interactions (PPIs)

The PPIs were analyzed using the Search Tool for the Retrieval of Interacting Genes (STRING) (v. 11.5) database [31] and the Cytoscape (v.3.9.1) software [32].

#### 2.9.4. Assessment of the Secondary and Tertiary Structure

The Self-Optimized Prediction Method with Alignment (SOPMA) server [33] was used to determine the secondary structural parameters of the desired protein. PSIPRED V.4.0 [34] was used to predict the secondary structure of the selected protein. Moreover, the tertiary structure of the protein was demonstrated using the Modeller program through the hidden Markov model–hidden Markov model–based protein prediction (HHpred) algorithms [35] and endorsed by the Procheck program along with the *Z*-score obtained from the Prosa Web server [36].

### 2.10. Isolation and Quantification of Bacterial Genomic DNA (gDNA)

There are three basic steps involved in the DNA extraction procedure: lysis, precipitation, and purification. In cytolysis, the nuclear material and the cell are ruptured open, thus liberating DNA. Various protocols are available for DNA extraction from bacteria. gDNA was separated from overnight culture using the Warlock gDNA Purification Kit (Promega) according to the instructions of the company [37].

### 2.11. Quantification of Bacterial DNA Using NanoDrop

The purity of DNA was measured using the Thermo Scientific NanoDrop 2000 c spectrophotometer, and the absorbance ratios were used: A260/280 and A260/230 nm to estimate the DNA purity—A260/280 nm for the protein impurity and A260/230 nm for the deionized salt and phenol impurity [38].

### 2.12. Designation of Primers

The gene coding for 16S rRNA is a molecular marker widely used for the molecular recognition of bacteria. We used universal primers to amplify the genes coding for RNA ribosomal 16S. Polymerase chain reaction (PCR) for the amplification of the 16S rRNA desired gene was executed using universal primers 27F (5⁣′-AGAGTTTGATCMTGGCTCAG-3⁣′) as forward and 1492R (5⁣′-CGGTTACCTTGTTACGACTT-3⁣′) as reverse primer [39].

#### 2.12.1. PCR Amplification of Genes Encoding 16S rRNA

PCR has become the most common technique used in medical and biological research laboratories for isolating and exponentially amplifying the specific region of DNA segments [40]. The PCR reaction was executed in a final volume of 20 *μ*L containing 7 *μ*L of sterile distilled water (dH2O), 2 *μ*L of DNA, 1 *μ*L of each sense and antisense primers, 10 *μ*L of master mix, and 1 *μ*L of T DNA. Amplification of the PCR was executed in Gene Atlas, model: G2, origin: Aztec, Japan, according to the following steps: preheat at 95°C for 3 min. Thirty cycles for each of the following steps: denaturation at 95°C for 30 s, annealing at 48°C for 30 s, initial elongation at 72°C for 1 min and 30 s, and a final elongation at 72°C for 5 min [41]. PCR products were visualized using the agarose gel electrophoresis technique.

### 2.13. Agarose Gel Electrophoresis of PCR Products

Agarose gel electrophoresis is an affordable, simple, rapid, and susceptible method widely employed for separating DNA and RNA molecules by size [42]. The PCR products were detected via 1.5% agarose gel with ethidium bromide.

### 2.14. Sequencing of Bacterial DNA and Phylogenetic Analysis

The Institute for Clinical and Laboratory Standards has published instructions on employing DNA sequencing to identify bacteria [43]. The 16S rRNA gene is the expected target used to identify bacterial isolates. The amplified PCR products of microbial gene fragments were purified and sequenced at 1^st^ Base Sequencing, Malaysia, using the automated sequencer ABI 3100 with Big Dye Terminator Kit v. 3.1. Primers 518F (5⁣′CCAGCAGCCGCGGTAATACG3⁣′) and 800R (5⁣′TACCAGGGTATCTAATCC3⁣′) were used for DNA sequencing studies [44]. The sequences or segments were found and differentiated from the National Center for Biotechnology Information (NCBI) database through Basic Local Alignment Search Tool (BLAST) searches (http://blast.ncbi.nlm.nih.gov/Blast.cgi). In this observation, sequences of type strains intimately related to the sequences of the isolates were searched. The sequences were aligned with ClustalW, and an evolutionary tree was built from the evolutionary distances by the maximum likelihood method with the software Molecular Evolutionary Genetics Analysis (MEGA X) [45].

### 2.15. Enzymatic Dehairing Activity

The dehairing capability was observed using the technique described [46]. The organism was grown in NB broth at 37°C for around 20 h and centrifuged at 6000 rpm for 15 min [47]. The obtained supernatant was filtered using 0.2 *μ*m pore filter paper, and this sample was used as the crude enzyme [48]. The enzyme was then purified using the following method from [49]. Further, goat skin/hid was cut into 2 × 2 cm pieces, gently washed with clean tap water, and then rinsed with dH2O to eliminate chemicals from the skin/hid, which may inhibit the enzyme activities during dehairing activities. 2(2 × 2 cm) skin pieces were inserted separately into 250 mL conical flasks. Then, 100 mL of crude enzyme solution was added sequentially and incubated at 37°C for 8 h. After incubation, pieces of skin were removed and peeled with a swab stick, and the dehairing capacity of the extracted crude protease enzyme was observed.

## 3. Results

### 3.1. Cultural Characteristics of Bacterial Isolates

**Figure 1:**
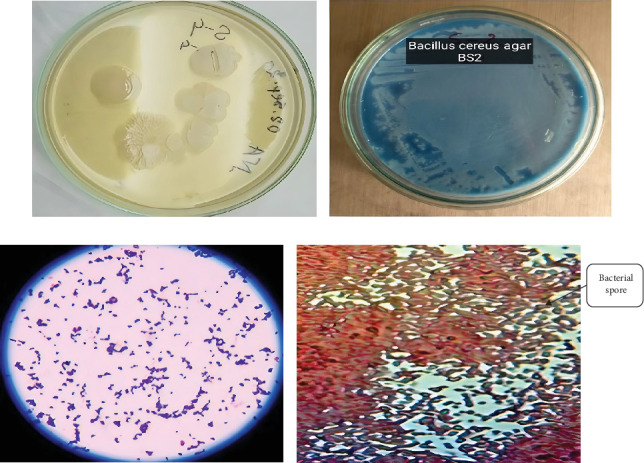
(a) Colony morphology of selected isolates (BS2), (b) pure culture of the isolate BS2 in *B*. *cereus* agar, (c) Gram staining of the selected isolate (BS2), and (d) spore staining of selected isolates (BS2).

In this study, four bacterial strains were isolated in culture media. Nutrient agar was selected to determine the best suitable media for securing massive growth of the isolated microbial strains. The nutrient agar medium was ideal for the enormous growth of BS1, BS2, BS3, and BS4. The isolated bacterial colonies were yellowish, whitish, or orange colored and only represented the BS2 results here and mentioned in Figure 1(a). The shape of the colonies was small, elongated, and circular.  Table 1 shows that most of them have been found to have an entire margin and were medium-sized and flat. Selective media such as *B. cereus* agar was also used to confirm *Bacillus* strains, initially confirmed as BS2 (Figure 1(b)).

### 3.2. Microscopic Observation

Morphological characteristics of isolated bacteria were analyzed using Gram staining and spore staining methods. The isolate BS2 retained the purple color of crystal violet stain, implying that they were Gram-positive and rod-shaped bacteria (Figure 1(c)).

Spore staining revealed that the isolate (BS2) was spore-forming (Figure 1(d)). Generally, bacteria produce spores in the growth medium under unfavorable conditions, usually bright green.  Table 2 determines the Gram reaction, spore, and shape of different isolates.

### 3.3. Biochemical and Phenotypic Characterization of the Bacterial Isolates

The biochemical characterization of the identified samples was performed according to *Bergey's 1994 Manual of Systematic Bacteriology* [49]. Several conventional microbiological and biochemical tests were carried out for the provisional identification of the isolates. Gram and spore staining confirmed that the isolate BS2 was a Gram-positive, rod-shaped, spore-forming bacterium. The isolates produced small and nonpigmented milky white colonies in the nutrient agar plates.  Table 3 represents the results of biochemical tests and other information, indicating that the organisms might be a member of the *Bacillus* family.

### 3.4. Proteolytic Activity of the Enzyme

The azocasein test measured the proteolytic activity of the enzyme. Here, azocasein is used as a substrate. The optical density of the sample is found to be 0.181 absorbance compared with the control, where no enzymatic reaction has occurred. The unit of enzyme activity of the sample is found to be 18.1. One unit of proteolytic activity is defined as the amount of enzyme that increases the absorbance to 0.01 at 440 nm.

### 3.5. Growth Profile and Protease Activity of the Organism at 37°C

**Figure 2:**
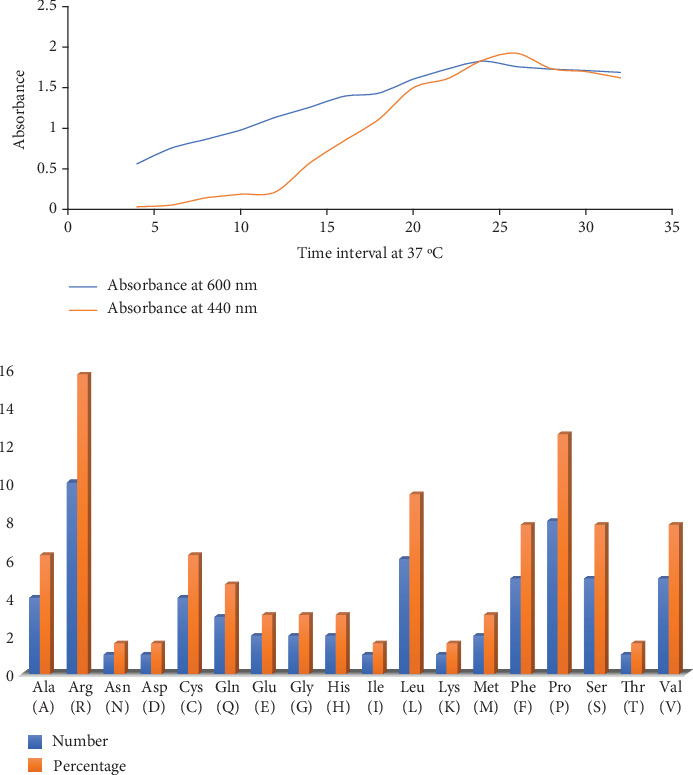
(a) Relation of growth with bacterial enzyme secretion at 37°C and enzyme activity and (b) amino acid composition.

Then, Kreger and Lockwood's methods (1981) were performed to measure the protease activity, as shown in Figure 2(a), and the absorbance was taken at 440 nm to measure the protease activity of the organism at 37°C.

The growth curve profile against the protease activity is illustrated in Table 4. In the initial growth stage, the basal level of extracellular protease increases with time, indicating a differential synthesis of the enzymes with growth time.

### 3.6. Bioinformatics Analysis for the Characterization

#### 3.6.1. Physicochemical Characterization

The appropriate criteria can be set when the physicochemical features of a protein therapy have been determined. The physicochemical qualities of the protein were established using the ProtParam tool of the ExPASy server, and their definition may be understood by analyzing the properties of the individual amino acids that make up the protein. The ExPASy server's ProtParam tool was used to determine the physicochemical characteristics of proteins by examining the properties of the amino acids. Molecular weight (MW) (7375.94 Da), aliphatic index (71.56), instability index (II, 80.63), Grand Average of Hydropathy (GRAVY) (−0.231), and isoelectric point (11.41) were anticipated using the ProtParam tool (Figure 2(b)). Moreover, the computationally estimated half-life of the founded proteins was demonstrated as about 30 h (mammalian reticulocytes, in vitro), > 20 h (yeast, in vivo), and > 10 h (*Escherichia coli*, in vivo).

#### 3.6.2. Subcellular Localization Determination

Protein subcellular localization regulates activity by determining which other molecules it can interact with. The PSORTb tool demonstrated the selected protein location as cytoplasmic, cytoplasmic membrane, cell wall, and extracellular properties, resulting in various localizations and activities. Moreover, the PSLpred server anticipated the protein as a periplasmic protein, and no transmembrane helix was found, as confirmed by the HMMTOP and DeepTMHMM servers.

#### 3.6.3. PPIs

**Figure 3:**
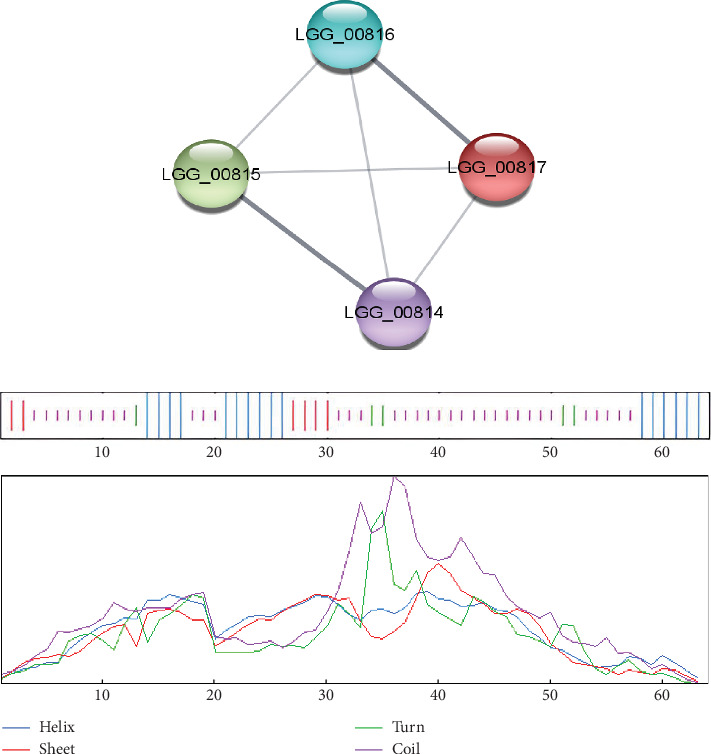
(a) The PPI networks were generated using the STRING database and Cytoscape software and (b) secondary structural parameter graphical representations.

The study of PPIs centers on understanding how cells and their systems function. These links provide a valuable framework for annotating functional, structural, and evolutionary characteristics and for filtering, assessing, and verifying functional genomics data of the proteins. The STRING and the Cytoscape software demonstrated the PPI of the selected protein, which is exhibited in Figure 3(a). The STRING database network stats identified four nodes, six edges, an average node degree of 3, an average local clustering coefficient of 1.0, and a PPI enrichment *p* value of 0.0522.

#### 3.6.4. Secondary and Tertiary Structural Assessment

**Figure 4:**
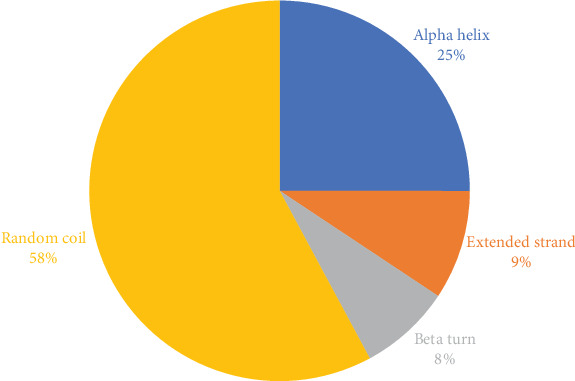
Structural characteristics of the selected protein.

The structure of the protein and its function are intricately intertwined. Protein function, framework, and interaction strongly connect to the secondary structural parameters, such as helices, coils, sheets, and turns. SOPMA predicted the secondary parameters with default parameters (Figure 3(b)). Secondary structures contain alpha helix (16, 25.00%), extended strand (6, 9.38%), beta-turn (5, 7.81%), and random coil (37, 57.81%) (Figure 4).

**Figure 5:**
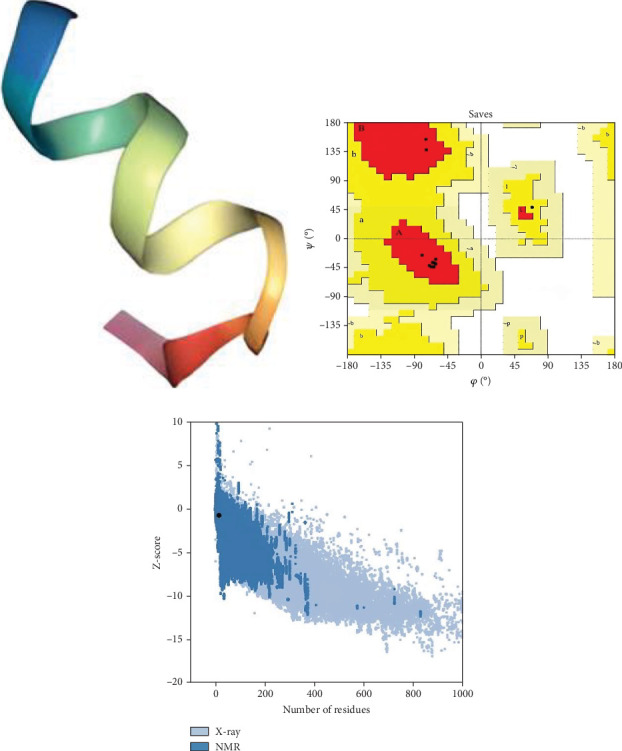
Three-dimensional structure. (a) The predicted structure, (b) the Ramachandran plot, and (c) the *Z*-score (−0.69).

Moreover, the HHpred features include searching for sequence similarity, building alignments, detecting characteristics in sequences, predicting their structures, and classifying sequences. The toolkit's versatility has established it as an invaluable asset in experimental biology and bioinformatics education [35]. The template for anticipating the three-dimensional structure was chosen based on the most suitable similarity (HHpred ID: 2K42_B) with the selected protein sequence. Furthermore, the predicted structure was validated by PROCHECK (SAVES V.6.0) and *Z*-score (Figure 5).

### 3.7. DNA Quantification

The DNA samples were quantified using the NanoDrop −2000 spectrophotometer. The purity level of DNA samples is analyzed and represented in Table 5.

### 3.8. PCR Amplification and Gel Electrophoresis

**Figure 6:**
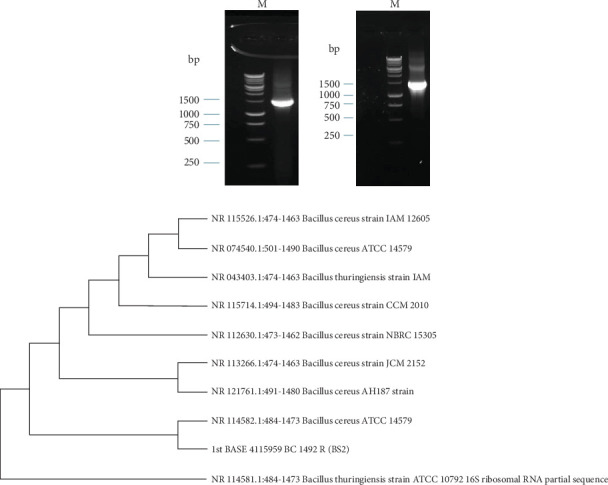
(a) Profiles of 27F and 1492R primers generated from bacteria; M denotes 1 kb DNA ladder. (b) Phylogenetic analysis of the isolate BS2 (*B. cereus*).

The PCR products were then analyzed using gel electrophoresis. The band of the 16S rRNA gene of BS2 in 1.5% gel electrophoresis is shown in Figure 6(a). The size of the PCR products was 1465 bp. We used a 1-kb ladder to determine the band size.

### 3.9. Molecular Identification Based on 16S rRNA Sequence of the Isolates

The PCR products were sent to first Base Sequencing Malaysia. 16S rRNA gene amplification and sequencing were reported to identify and characterize *Bacillus* sp. [50]. The BS2 (*B. cereus*) sequence was compared with other bacteria species to check the similarity of the 16S rRNA gene and their phylogenetic lineage.  Figure 6(b) represents the phylogenetic analysis of nucleotide sequences based on the 16s rRNA by BLAST; it was revealed most closely that the isolate BS2 was identified as *B. cereus*.

### 3.10. Dehairing Capacity of Crude Enzyme of Isolated Bacteria

To observe the dehairing capacity of BS2, the fresh hairy hide of 2 × 2 cm size was immersed completely into 10 mL supernatant and incubated at 37°C. Treated skins and controls exhibited visible significant differences after 9 h of incubation. No color change was found, although corroded areas were observed in enzyme-treated skins. After the treatment, the enzyme emerges quickly when the hairs get pulled with forceps. After 9 h of incubation, scraping can easily remove intact hairs from the skin. The experiment was repeated three times, and the result was reproducible.

**Figure 7:**
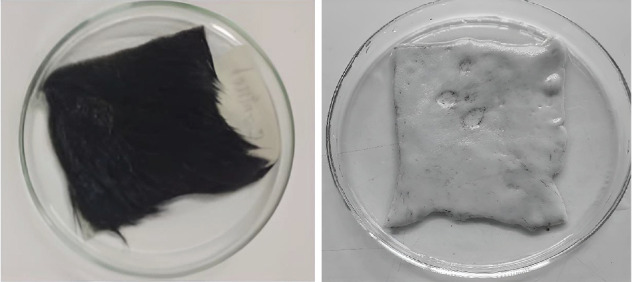
Enzymatic dehairing of goat hides (BS2).

Hair splitting was not noticed in controls, even with the mechanical action of forceps. This result was far better than the other bacteria created by dehairing. dH2O was used as a control. So, the use of microbial enzymes as an alternative technology to conventional methods highlights the importance of these enzymes in reducing pollution loads. The enzymatic test result is presented in Figure 7.

#### 3.10.1. Comparison of Dehairing Capability of *B. cereus* MW703982 With Another Bacterium

Table 6 demonstrates the dehairing capacity of the protease produced by our strain. Compared to the other bacterial proteases, our bacterial protease enzyme dehaired much faster than the other three.

## 4. Discussion

Protease enzymes significantly reduce the usage of bleaching agents, alkalis, acids, and different disease-causing chemicals in leather processing factories. They are also responsible for low environmental pollution and consumption of less water [52]. This study found rod-shaped, spore-forming, Gram-positive bacterial species from tannery waste samples that showed positive reactions with most biochemical tests without lactose and sucrose fermentation. Likewise, Zhao et al. [53] exhibited similar findings from dairy products in China. This bacterial species shows positive results with the azocasein test and demonstrates proteolytic solid activity.

This study identified two bacterial strains that can produce extracellular protease enzymes and quickly dehair goat hide, showing potential applications in tannery industries. We attempted to identify and characterize the organisms because their dehairing activity was rapid and effective, and the grade of dehaired leather was more trustworthy than typical chemical dehaired hide. Based on morphological and biochemical characterization, it was found that the isolated organisms exhibited high homology to *Bacillus* sp. Further investigation by BLAST analysis of 16S rRNA sequence and evolutionary analysis of that sequence collectively attuned the isolate BS2 as a member of the *B. cereus* family. Evolutionary analysis showed that *B. cereus* (BS2) is closely connected with *B. thuringiensis*; this could be because these bacterial species are members of the same family, are rod-shaped, which produce spores; these are autonomous anaerobes and cause a variety of gastrointestinal disorders [54]. However, there are no similarities between their genomes and genetic materials [55].

Similarly, Sundararajan, Kannan, and Chittibabu [56] isolated the bacterial strain from the soil, and after 16S rRNA sequencing, phylogenetic tree analysis was conducted to determine the isolated strain VITSN04 and confirmed as *B. cereus*. Likewise, Rahman, Islam, and Zohora [3] isolated an organism from tannery soil and identified *B. subtilis* based on its morphological and physiological characteristics, biochemical tests, API system, and 16S rRNA sequencing. Previously, various studies were conducted on the isolation, purification, and characterization of subcellular protease enzymes producing *B. subtilis*, which is compatible with our study [57]. This study provided the potential use of protease enzymes in leather processing to remove pollution-causing chemicals such as sodium, lime, and solvents [58]. Their investigation of a novel keratinase from *B. subtilis* can alter sodium sulfide in the dehairing process and produce hair-removing enzymes without breaking the fibrous collagen of the dermis.

This investigation for removing hair from goat skin undergoes the process without adding any additive of hazardous chemicals, thus making it a potential candidate for possible biotechnological application towards the development of intelligent, clean, pollution-free, green leather processing technology compared to conventional chemical methods. Similarly, the crude enzyme isolated from all the bacteria was able to remove hair from goat hide, but their effectiveness differed. We observed that the isolated crude enzyme of *B. cereus* (BS2) was more effective on goat skin than other bacteria at 37°C. Proteases that showed suitable activity in alkaline conditions (pH: 8–12) and are stable at alkaline pH are potential candidates for extracting hides [59]. However, this biological leather remover has several challenges in gaining popularity. For example, the main barrier to using protease enzymes is improving enzyme effectiveness and durability for marketing [60]. Other obstacles are substrate specificity and thermostability, and environmentalists have made manufacturing and chemical-based companies their main targets for their fight against contamination; the leather sector is likewise not exempt because chemicals are used more efficiently than protease enzymes; these factors make it difficult for the leather sector to embrace protease enzymes [61]. This study found protease enzymes producing *B. cereus* species with significant proteolytic activity, effectively removing hair from goat skin. Hence, our findings are crucial for leather industries to leather processing and demonstrate the potential ability to reduce the disease-causing effects (cancer, cardiovascular, and stroke infectious diseases) on leather workers and environmental pollution.

## 5. Conclusion

The leather industries play an essential role in Bangladesh's economy due to its enormous potential for employment, growth, and export. Meanwhile, it poses serious environmental threats by discharging liquid effluents and solid wastes directly into surrounding low-lying areas without proper treatment. Tannery waste is a mixture of various substrates. Thus, it is an excellent enrichment medium for cultivating numerous microorganisms. Bacteria in this environment are metabolically active, which produces multiple enzymes and bioactive compounds compared to other environmental conditions. This study identified bacterial isolates from tannery soil based on morphological, cultural, biochemical, and molecular characteristics. This study revealed that the isolated enzymes from *B. cereus* of tannery soil could substitute hazardous chemicals for leather dehairing, which can control environmental pollution. Further investigation will need to characterize the enzyme properties applicable to ecofriendly tools in the leather processing industry.

## Figures and Tables

**Table 1:** Cultural characteristics of bacterial strains.

**Strains**	**Color on nutrient agar**	**Configuration**	**Margin**	**Elevation**
BS1	Yellow	Circular	Entire	Convex
BS2	Off-white	Circular	Entire	Convex
BS3	Mucoid	Circular	Entire	Slightly raised
BS4	White	Circular lobate	Irregular	Flat

**Table 2:** Microscopic characterization of different isolates.

**Strains**	**Gram staining**	**Spore staining**
BS1	Gram negative	Nonspore forming
BS2	Gram positive	Spore-forming and rod-shaped
BS3	Gram negative	Nonspore forming
BS4	Gram positive	Very low spore forming

**Table 3:** Biochemical and morphological features of the desired bacterial isolate.

**Parameters**	**Bacterial isolate BS2 (*Bacillus cereus*)**
Cell shape	Rod
Cell arrangement	Short chain
Colonial pigmentation	Gray white
Gram staining	+ve
Endospore staining	+ve
Motility test	+ve
Catalase test	+ve
Indole production	+ve
Starch hydrolysis	+ve
Gelatin hydrolysis	−ve
Casein hydrolysis	+ve
Urease test	+ve
Sucrose fermentation	+ve

*Note:* Key: +ve = positive; −ve = negative.

**Table 4:** Growth profile and protease activity of the bacteria isolate BS2 at 37°C.

**Time at hours**	**Absorbance at 600 nm**	**Absorbance at 440 nm**
4	0.561	0.031
6	0.755	0.053
8	0.863	0.142
10	0.977	0.186
12	1.133	0.213
14	1.258	0.569
16	1.394	0.845
18	1.433	1.109
20	1.605	1.498
22	1.732	1.613
24	1.827	1.837
26	1.76	1.924
28	1.729	1.736
30	1.712	1.699
32	1.689	1.622

**Table 5:** Results of DNA quantification in NanoDrop.

**Sample**	**Sample type**	**Unit**	**A260 (abs)**	**A280 (abs)**	**260/230**	**260/280**
BS2	DNA	ng/*μ*L	11.757	5.415	2.14	2.17

**Table 6:** The comparison of the dehairing capability of *B. cereus* MW703982 with other species of bacterium [51].

**Bacterial species**	**Time of incubation for dehairing**	**Change of color of leather**
*B. cereus* MW703982	9 h	Color unchanged
*Vibrio* sp. kr2	24 h	Color unchanged
*Flavobacterium* sp. kr6	24 h	Color unchanged
*Bacillus* sp. kr10	14 h	Color unchanged

## Data Availability

The data used to support this study's findings are available from the submitting or corresponding authors upon request.
